# Department of Hygiene at the University of Pennsylvania

**Published:** 1890-01

**Authors:** 


### Department of Hygiene at the University of Pennsylvania.

A very important announcement was made November the 11, in regard to the University of Pennsylvania, namely, that Dr. John S. Billings has, with the approval of the Secretary of War and of the Surgeon General, accepted the position of Medical Director of the University Hospital, to which he was recently elected, and that the duties of this new position will be so arranged as not to interfere with his duties as Medical Director of the Army at the Surgeon General's office.

It is also announced that the University of Pennsylvania is soon to have a new laboratory of hygiene to cost about $200,000; and that $100,000 have been already collected for this purpose. The Department of Hygiene has been under tthe supervision of Dr. Samuel G. Dixon since the death of Dr. N. A. Randolph, who was recently one of the editors of the *Medical and Surgical Reporter.*

It was rumored at first that Dr. Billings was to supersede Dr. Dixon, but the Provost of the University promptly denied this rumor and stated that Dr. Dixon was still Professor of Hygiene in the University and in charge of the Laboratory of Hygiene, which has been equipped through his exertions and liberality.

It does not yet appear just what Dr. Billings will do at the University, but the propability is that he will be at the head of Department of Hygiene. For this he is abundantly fitted, as he is recognized as an authority upon the subjects of hygiene and hospital connection and administratian. The elaboration of the plans for the construction of the Johns Hopkins Hospital, and his coming will be a valuable accession to the teaching force of the University of Pennsylvania.—*Med. and Surgical Reporter.*

The above is encouraging, especially to those who have asked,

Why is sanitary science not made one of the prominent branches in the course of instruction in all medical colleges? It is certainly more desirable and more important to conserve the health of the people than to cure disease.

The physician should be the conservator of the public health; he should above all devote an intelligent ability to diverting or destroying all disease-producing influences or agencies. And he ought to be educated with this in view, and then he should be, by the proper authority, placed in a position to use his ability for the greatest good.

This would be a revolution from former customs and modes of procedure, but change and progress is the order of the day in this time of the world's history. May the time soon come when sanitary science will be one of the leading branches in the course of instruction in every medical and dental college in the country.

[Ed. Register.

